# Risk of Chronic Low Back Pain Among Parturients Who Undergo Cesarean Delivery With Neuraxial Anesthesia: A Nationwide Population-Based Retrospective Cohort Study

**DOI:** 10.1097/MD.0000000000003468

**Published:** 2016-04-22

**Authors:** Yuan-Yi Chia, Yuan Lo, Yan-Bo Chen, Chun-Peng Liu, Wei-Chun Huang, Chun-Hsien Wen

**Affiliations:** From the Department of Anesthesiology (Y-YC, YL, Y-BC, C-HW), Kaohsiung Veterans General Hospital, Kaohsiung, Taiwan; Critical Care Center and Cardiovascular Medical Center (C-PL, W-CH), Kaohsiung Veterans General Hospital, Kaohsiung, Taiwan; School of Medicine (W-CH, C-PL), National Yang-Ming University, Taipei, Taiwan; and Department of Physical Therapy (W-CH), Fooyin University, Kaohsiung, Taiwan.

## Abstract

To investigate the risk of chronic low back pain (LBP) in parturients undergoing cesarean delivery (CD) with neuraxial anesthesia (NA).

LBP is common during pregnancy and also after delivery, but its etiology is poorly understood. Previous studies that investigated the correlation between epidural labor analgesia and chronic low back pain were inconclusive. These studies lacked objective diagnostic criteria for LBP and did not exclude possible confounders. We performed this nationwide population-based retrospective cohort study to explore the relationship between CD with NA and subsequent LBP.

From the Taiwan National Health Insurance Research Database (NHIRD), we identified all primiparas who had given birth between January 1, 2000 and December 31, 2013. Using the International Classification of Diseases, Ninth Revision, Clinical Modification (ICD-9-CM) procedure codes, we identified the women who had vaginal delivery (VD) and those who had CD. The mode of anesthesia was ascertained by the NHI codes. Multivariable logistic regression was used to estimate the odds of postpartum LBP in women undergoing CD with NA compared with those having VD. The outcome was a diagnosis of LBP according to the first ICD-9-CM diagnosis code. The patients were observed for 3 years after delivery or until diagnosis of postpartum LBP, withdrawal from the NHI system, death, or December 31, 2013.

Of the 61,027 primiparas who underwent delivery during the observation period, 40,057 were eligible for inclusion in the study. Of these women, 27,097 (67.6%) received VD, 8662 (21.6%) received CD with spinal anesthesia, and 4298 (10.7%) received CD with epidural anesthesia (EA). Women who received CD with EA were found to have higher risk of LBP than did women who received VD, with the adjusted OR being 1.26 (95% CI: 1.17–1.34).

CD with EA might increase the risk of subsequent chronic LBP.

## INTRODUCTION

Low back pain (LBP) is common during pregnancy and also after delivery. According to most studies, at least half of the pregnant population is affected.^1–3^ Persistence of LBP for at 6 months after delivery has been reported in 5% to 40% of patients.^4–6^ The etiology is poorly understood.^7^ Many parturients and their obstetricians believe that spinal anesthesia will cause LBP.^8^ One study that compared the incidence of postpartum LBP following natural childbirth with that after cesarean delivery (CD) with spinal anesthesia (SA) found no difference between the 2 groups.^8^ However, the sample size was small, and the authors did not compare CD with other methods of anesthesia, such as epidural anesthesia (EA) and general anesthesia (GA), with vaginal delivery (VD). A retrospective study of 11,701 women found that chronic LBP occurred after delivery more frequently in women who had received EA during labor than in women who had not (19% vs 11%) and concluded that there was a causal relationship between EA and back pain.^9^ However, a series of prospective studies by these authors and others found no correlation between epidural labor analgesia and increased incidence of chronic LBP.^10–16^ Most of these studies used subjective questionnaires as the evaluation method, examined single hospital cases, and did not exclude variables that may have confounded the results.

To date, national-level data and large-scale studies on the incidence of postpartum LBP in parturients with VD and CD with neuraxial anesthesia (NA) are lacking. To address this gap, and on the basis of the hypothesis that parturients with NA have a higher risk of developing LBP, we designed this nationwide population-based retrospective cohort study to explore the link between NA and LBP.

## METHODS

### Data Sources

The data for this study were collected from the Taiwan National Health Insurance Research Database (NHIRD) for the period 2000 through 2013. This dataset, organized and managed by the Taiwan National Health Research Institutes, has been collected by the Taiwan National Health Insurance Program since 1995. The program covers approximately 99% of Taiwan residents and has contracts with 97% of medical providers nationwide.^17^ The database includes the entire patient registry and claims data from this health insurance system, with information ranging from demographic data to detailed orders from ambulatory and inpatient care. All data are deidentified through encryption of the identification codes of patients and medical facilities to preserve patient anonymity. Personal information, such as body weight and height, results of laboratory tests, and details of lifestyle factors, is not available in the NHIRD.^18^ To verify the accuracy of the diagnoses recorded in the database, the Taiwan Bureau of National Health Insurance randomly interviews patients and reviews the charts of 1 per 100 ambulatory and 1 per 20 inpatient claims.^19^ The NHIRD has been extensively used in epidemiologic studies in Taiwan.^20–22^ The data used in this study were retrieved from the Longitudinal Health Insurance Database 2000 (LHID 2000), a subset of the NHIRD. The LHID 2000 is a dataset released by the NHRI that contains all original claims data for 1 million randomly selected beneficiaries in the 2000 Registry of Beneficiaries.

### Ethics Statement

The institutional review board of Kaohsiung Veterans General Hospital approved this study. Written consent from the patients was not obtained because the NHI dataset consists of deidentified secondary data used for research purposes, and the institutional review board of Kaohsiung Veterans General Hospital issued a formal written waiver of the need for consent.

### Study Population

Using the data in the LHID 2000, we identified 61,027 primiparas who had given birth (International Classification of Diseases, Ninth Revision, Clinical Modification [ICD-9-CM] procedure codes 72, 73, 74) between January 1, 2000 and December 31, 2013. We excluded patients (n = 16,388) who were diagnosed with LBP (ICD-9-CM codes 721.3, 722.1, 724.02, 724.09, 724.2, 724.3, 724.5, 724.9) before the day of delivery to accurately examine the sequential relationship between delivery and LBP. We also excluded patients (n = 4582) who had extreme values of maternal age (i.e., age ≥ 50 years), and missing data. Ultimately, 40,057 women were included in the study; of these women, 27,097 had VD (ICD-9-CM procedure codes 72, 73), 8662 had CD with SA (NHI Codes 96007C, 96008C), and 4298 had CD with EA (NHI Codes 96005C, 96006C).

### Variables of Interest

In this study, the independent variables of interest were VD, CD with SA, and CD with EA. The endpoint was outpatient treatment of LBP or hospitalization for treatment of any type of LBP (ICD-9-CM codes 721.3, 722.1,724.02, 724.09, 724.2, 724.3, 724.5, 724.9) after delivery. To ensure diagnostic validity and patient homogeneity, only patients who were diagnosed with LBP according to the first diagnosis code were included in the study group. The index date was defined as the date of delivery of the patients enrolled in our study.

To assess the independent effects of VD and the different modes of NA for CD on LBP, we adjusted for several possible confounding variables, namely the patient's age in years, multiple gestation (ICD-9-CM code 651), diabetes mellitus (ICD-9-CM code 250), obesity (ICD-9-CM codes 278.00, 278.01), pregnancy-related hypertension (ICD-9-CM code 642.3), complicated obstetric conditions (including preeclampsia [ICD-9-CM codes 642.4, 642.5] and eclampsia [ICD-9-CM code 642.6]), urinary tract infection (ICD-9-CM code 599.0), urinary tract stone (including renal stone [ICD-9-CM code 592.0] and ureteral stone [ICD-9-CM code 592.1]). The patients were observed for 3 years after delivery or until diagnosis of postpartum LBP, withdrawal from the NHI system, death, or December 31, 2013.

### Statistical Analysis

Continuous data were presented as means (± standard deviation). Categorical data were presented as numbers and percentages. The incidence of newly diagnosed LBP in the VD patients and the CD with NA patients was calculated, and the F-test in analysis of variance (ANOVA) and the chi-square test were used to examine the differences in the characteristics between the 2 groups. Univariate logistic regression was performed to estimate the odds of LBP in patients undergoing CD with NA compared with patients undergoing VD. Multivariable logistic regression was used to estimate the odds of LBP in CD with NA patients after adjusting for the various confounding variables enumerated earlier.

We used SAS for Windows, Version 9.4 (SAS Institute, Cary, NC) for data extraction, computation, linkage, processing, and sampling. All other statistical analyses were performed using SPSS for Windows, Version 18 (IBM, Armonk, NY). *P* < 0.05 was considered to indicate a statistically significant relationship.

## RESULTS

Among the 40,057 primiparas who underwent delivery during the observation period, 27,097 (67.6%) received VD, 8662 (21.6%) received CD with SA, and 4298 (10.7%) received CD with EA. The numbers of primiparas who had LBP during overall follow-up duration were 8561 (31.59%) in VD, 2756 (31.82%) in CD with SA, and 1536 (35.74%) in CD with EA. Comparison of the sample characteristics between the VD patients and the CD with SA or EA patients revealed significant differences between the groups (Table 1). Compared with the VD patients, the CD with SA or EA patients were slightly older and included a higher number of parturients with diabetes, obesity, pregnancy-related hypertension, complicated obstetric conditions (including preeclampsia and eclampsia), urinary tract infection, and urinary tract stone (including renal stone and ureteral stone). These potentially confounding variables were adjusted for in the multivariate logistic regression models.

**TABLE 1:**
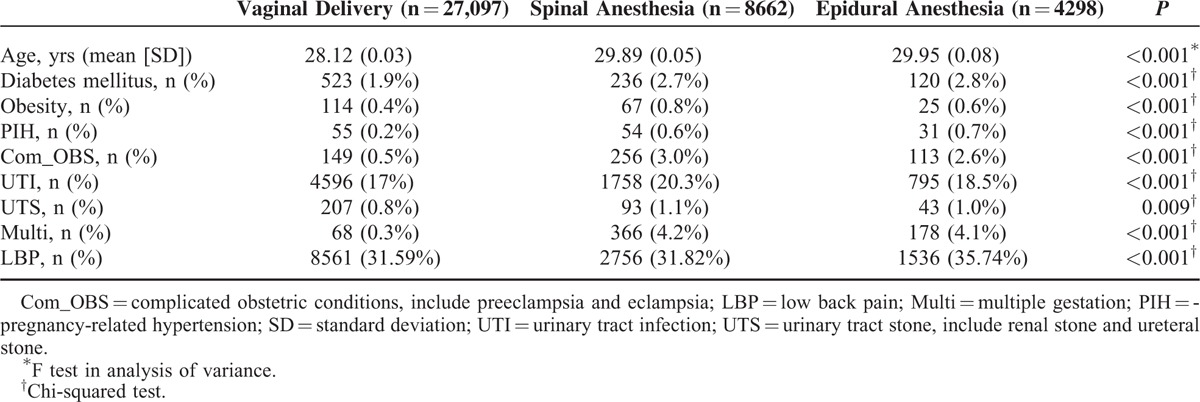
Comparison of Patient Characteristics Among the Vaginal Delivery and Caesarean Delivery With Spinal Anesthesia or Epidural Anesthesia

Logistic regression analysis was used to test the association between NA and postpartum LBP. We found no significant differences between the VD patients and the CD with NA patients for LBP occurring within 3 months of delivery (Table 2). A subanalysis based on the duration of follow-up revealed that the risk of newly diagnosed postpartum LBP was significantly elevated in the CD with EA group compared with the VD group (OR 1.20, 95% CI: 1.13–1.29; *P* < 0.001) (Table 2).

**TABLE 2:**
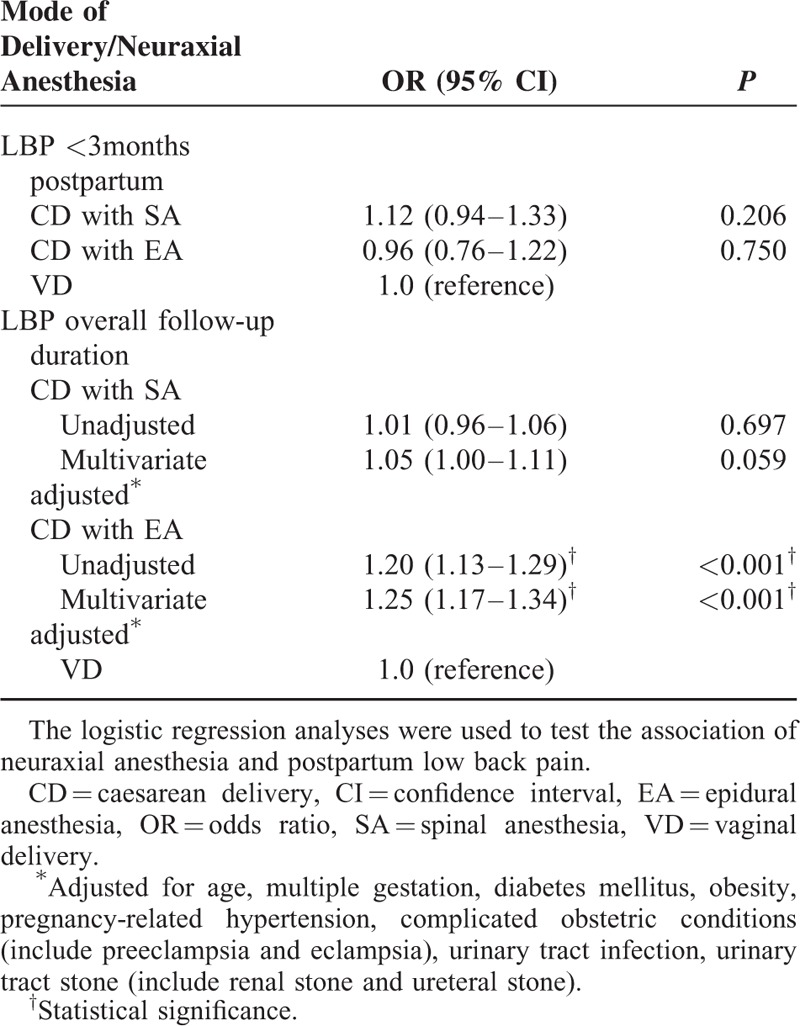
Mode of Neuraxial Anesthesia in Predicting Postpartum Low Back Pain

After adjustment for the potential confounders, we found that patients who received CD with EA had a significantly higher risk of postpartum LBP comparing with the VD parturients (OR 1.26, 95% CI: 1.17–1.34; *P* < 0.001) (Table 2). In the multivariable logistic regression analysis model, we found that age (OR 1.02, 95% CI: 1.02–1.03; *P* < 0.001) and urinary tract infection (OR 1.17, 95% CI: 1.11–1.24; *P* < 0.001) were both independently associated with an increased risk for postpartum LBP (Table 3).

**TABLE 3:**
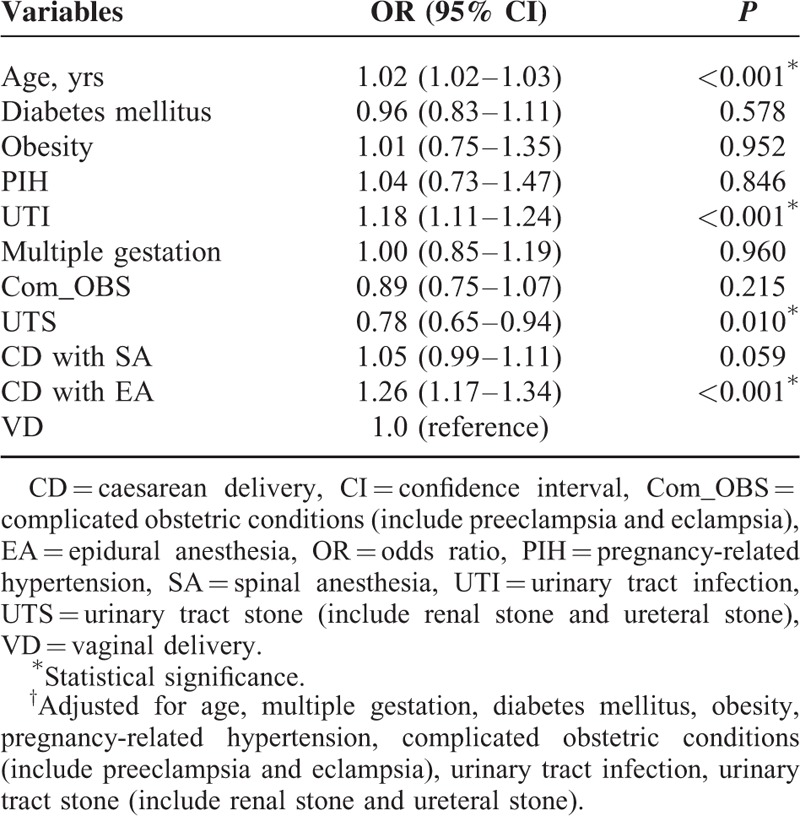
Multivariate Logistic Regression Analyses of Potential Predictors for Postpartum Low Back Pain^†^

## DISCUSSION

According to a review of relevant literature, this is the first large, nationwide population-based cohort study to investigate the risk of postpartum LBP in primiparas undergoing CD with NA compared with those undergoing VD. Our study yielded an adjusted OR of 1.26 for LBP in the CD with EA group compared with the VD group (Table 2). Greater age, multiple gestation, diabetes mellitus, obesity, pregnancy-related hypertension, complicated obstetric conditions (including preeclampsia and eclampsia), urinary tract infection, and urinary tract stone (including renal stone and ureteral stone) were all more prevalent in the parturients with CD with NA than in the parturients with VD (Table 1); this is consistent with the results of previous studies.^23–27^

Our results indicate that CD with EA may be a risk factor for subsequent LBP. There are several possible explanations for this. First, as MacArthur et al proposed, the most plausible hypothesis is that the origin of the problem is postural. Stressed positions can occur in normal labor and independently give rise to subsequent backache. Such postural problems can be aggravated by muscular relaxation and abolition of pain associated with epidural anesthesia.^9^ The nonselective nerve block induced by the epidural administration of a local anesthetic causes muscular relaxation in the lower back and legs, leading to immobility and long periods in stressed positions. In addition, movement under EA generally requires assistance, and a woman can remain in a potentially damaging position for several hours without complaining of any discomfort. Thus, epidural-related back pain could be initiated by the loss of normal joint protective reflexes due to anesthesia, leading to prolonged maintenance of poor posture and stressed positions during labor.^28^ Such stressed positions under EA may damage the back and lead to chronic LBP. The authors also found that many symptoms began in the first week after delivery, but in some women, LBP did not appear until several weeks after delivery, although it was still associated with EA. This implicates initial stresses in the development of LBP, with some cases requiring additional postpartum triggers to precipitate symptoms.^29^ A prospective cohort study with follow-up at different time after delivery analyzing women who underwent EA during labor and delivery found that the association between EA and new onset postpartum LBP was inconsistent over time: the relative risk for LBP (epidural vs nonepidural) only on day 1 (52% vs 39%, adjusted relative risk 2.05) and at 6 weeks (15% vs 7%, adjusted relative risk 3.17) with parity, delivery, ethnicity, and weight adjusted. It suggested that women underwent EA during delivery had an increased incidence of LBP on the first day possibly because of local musculoligamentous trauma associated with insertion of the needle.^12^

The association between EA and LBP has been hypothesized that poor posture during labor and delivery because of effective analgesia, muscular relaxation, immobility, and stressed posture results in primarily postural pain.^9^ But it could be arguable that the analgesia with EA usually lasts <12 hours, other more plausible risk factors shall be considered. The etiology is multifactorial. Enormously physical and physiological changes during pregnancy and after delivery such as lumbar lordosis, center of gravity rise and fall, loss of abdominal muscle support resulted in intense stretch on the lower back. Maternal workload such as repetitively lifting baby in bent forward and twisted positions,^12,30,31^ heavy physical work and even tedious housework, subjective perception of physical strains, and physical exertion are, particularly, regarded as the assumed cause by patients with LBP compared to patients with other origin chronic pain^32^; these all contribute to trigger LBP after delivery. For most women, pain resolves spontaneously, or gets improved with medical attention and simple treatment; very few of patients with sciatica and neurologic claudication might need timely surgical intervention.^31,33^ Other conditions like antenatal complaints of headaches or abdominal pain, endurance of back flexors, musculoskeletal subsystem imbalance, hormonal and vascular factors, and predisposing factors like greater weight and shorter stature, age, marital state, and socioeconomic status are also recognized as risk factors of parturients LBP thus shall be analysed.^9,33,34^

A second explanation for the pathogenesis of LBP is the presence of an epidural hematoma. Back pain is the usual presenting symptom in cases of epidural hematomas, either spontaneous in origin or associated with spinal or epidural procedures.^35–37^ Specific nociceptors are found in intramuscular^38^ and periosteal^39^ tissues, and one cause of epidural-related back pain may be the activation of these nociceptors by the small hematomas associated with epidural needle insertion.^28^

Our study results revealed that women with urinary tract infection may have an increased risk of LBP (Table 3). This is consistent with previous studies.^40^ We also found that older age is an independent risk factor for LBP (Table 3). These findings are inconsistent with some earlier studies,^6,9,33,41,42^ but consistent with others.^43,44^ Both younger and older ages have been reported to be associated with an increased risk of persistent LBP. In younger women, this is possibly due to the more pronounced collagen laxity as a result of higher sensitivity to the effects of hormones such as relaxin and estrogens.^41,42^ Our results suggest that older women are more likely to have LBP than younger women. This is unsurprising because older women are more likely to have degenerative changes in the spine. Our results are compatible with our empirical observation that older patients tend to have more likely to have pain even in the nonpregnant state.

The strengths of our study are the large sample size, consideration of confounding factors during analysis, and the exclusion of women with a history of LBP preceding pregnancy. In addition, our study design included an unbiased participant selection process. Because participation in the NHI is mandatory and all residents of Taiwan can access health care with low copayments, referral biases are low, and follow-up compliance is high.

However, our study had certain limitations. First, the NHIRD does not contain detailed information on parturients who received epidural analgesia for painless labor during VD, which is a self-paid item. Therefore, the prevalence of epidural painless labor in Taiwan could not be determined. According to previous reports, the prevalence of epidural painless labor is between 17.47% and 60.8% in urban medical centers in Taiwan.^45–48^ This is lower than that in many developed countries, such as the United States and the countries of the European Union, where the prevalence ranges between 23.6% and 78%.^49–51^ Moreover, we believe that the prevalence in the rural areas of Taiwan is lower than in the urban areas because of the paucity of anesthesiologists in the rural areas. During analysis, we did not exclude epidural painless labor in the VD group, because this would most likely have resulted in underestimation of the association between EA and LBP. Second, the diagnosis of LBP was identified using the ICD-9 codes from the database, and we may have underestimated the prevalence of LBP because only patients seeking medical evaluation could be identified; however, this may also result in underestimation of the association between EA and LBP. Third, although the data we obtained on EA and LBP were highly reliable, the diagnoses in NHI claims are primarily for administrative billing and are not scientifically verified.

## CONCLUSIONS

Our nationwide population-based retrospective cohort study provides further evidence of an increased risk of LBP among parturients receiving CD with EA. LBP is a treatable illness that has a substantial impact on the quality of life for parturients after delivery. Therefore, clinicians should be alerted to the possibility that parturients receiving CD with EA develop LBP.

## References

[1] Kristiansson P Svardsudd K von Schoultz B Back pain during pregnancy: a prospective study. *Spine* 1996; 21:702–709. 8882692 10.1097/00007632-199603150-00008

[2] Mogren IM Pohjanen AI Low back pain and pelvic pain during pregnancy: prevalence and risk factors. *Spine* 2005; 30:983–991. 15834344 10.1097/01.brs.0000158957.42198.8e

[3] Ostgaard HC Andersson GB Karlsson K Prevalence of back pain in pregnancy. *Spine* 1991; 16:549–552. 1828912 10.1097/00007632-199105000-00011

[4] Ostgaard HC Andersson GB Postpartum low-back pain. *Spine* 1992; 17:53–55. 1531555 10.1097/00007632-199201000-00008

[5] Ostgaard HC Roos-Hansson E Zetherstrom G Regression of back and posterior pelvic pain after pregnancy. *Spine* 1996; 21:2777–2780. 8979325 10.1097/00007632-199612010-00013

[6] Turgut F Turgut M Cetinsahin M A prospective study of persistent back pain after pregnancy. *Eur J Obstet Gynecol Reprod Biol* 1998; 80:45–48. 9758258 10.1016/s0301-2115(98)00080-3

[7] Mogren IM Does caesarean section negatively influence the post-partum prognosis of low back pain and pelvic pain during pregnancy? *Eur Spine J* 2007; 16:115–121. 16676151 10.1007/s00586-006-0098-8 PMC2198879

[8] Wang CH Cheng KW Neoh CA Comparison of the incidence of postpartum low back pain in natural childbirth and cesarean section with spinal anesthesia. *Acta Anaesthesiologica Sin* 1994; 32:243–246. 7894920

[9] MacArthur C Lewis M Knox EG Epidural anaesthesia and long term backache after childbirth. *BMJ* 1990; 301:9–12. 2143425 10.1136/bmj.301.6742.9 PMC1663375

[10] Breen TW Ransil BJ Groves PA Factors associated with back pain after childbirth. *Anesthesiology* 1994; 81:29–34. 8042807 10.1097/00000542-199407000-00006

[11] Russell R Dundas R Reynolds F Long term backache after childbirth: prospective search for causative factors. *BMJ* 1996; 312:1384–1388. 8646094 10.1136/bmj.312.7043.1384a PMC2351110

[12] Macarthur A Macarthur C Weeks S Epidural anaesthesia and low back pain after delivery: a prospective cohort study. *BMJ* 1995; 311:1336–1339. 7496283 10.1136/bmj.311.7016.1336 PMC2551244

[13] Macarthur AJ Macarthur C Weeks SK Is epidural anesthesia in labor associated with chronic low back pain? A prospective cohort study. *Anesth Analg* 1997; 85:1066–1070. 9356101 10.1097/00000539-199711000-00019

[14] Howell CJ Kidd C Roberts W A randomised controlled trial of epidural compared with non-epidural analgesia in labour. *BJOG* 2001; 108:27–33. 11213000 10.1111/j.1471-0528.2001.00012.x

[15] Howell CJ Dean T Lucking L Randomised study of long term outcome after epidural versus non-epidural analgesia during labour. *BMJ* 2002; 325:357. 12183305 10.1136/bmj.325.7360.357 PMC117883

[16] Loughnan BA Carli F Romney M Epidural analgesia and backache: a randomized controlled comparison with intramuscular meperidine for analgesia during labour. *Br J Anaesth* 2002; 89:466–472. 12402727

[17] Chen LF Chang CM Huang CY Home-based hospice care reduces end-of-life expenditure in Taiwan: a population-based study. *Medicine* 2015; 94:e1613. 26402827 10.1097/MD.0000000000001613 PMC4635767

[18] Wang YP Chen YT Tsai CF Short-term use of serotonin reuptake inhibitors and risk of upper gastrointestinal bleeding. *Am J Psychiatry* 2014; 171:54–61. 24030313 10.1176/appi.ajp.2013.12111467

[19] Tseng CH Mortality and causes of death in a national sample of diabetic patients in Taiwan. *Diabetes Care* 2004; 27:1605–1609. 15220235 10.2337/diacare.27.7.1605

[20] Wu CS Shau WY Chan HY Utilization of antidepressants in Taiwan: a nationwide population-based survey from 2000 to 2009. *Pharmacoepidemiol Drug Saf* 2012; 21:980–988. 22511574 10.1002/pds.3255

[21] Chang CH Lin JW Chen HC Non-steroidal anti-inflammatory drugs and risk of lower gastrointestinal adverse events: a nationwide study in Taiwan. *Gut* 2011; 60:1372–1378. 21415083 10.1136/gut.2010.229906

[22] Wu CS Wang SC Cheng YC Association of cerebrovascular events with antidepressant use: a case-crossover study. *Am J Psychiatr* 2011; 168:511–521. 21406464 10.1176/appi.ajp.2010.10071064

[23] Magann EF Ounpraseuth ST Miller CD Maternal and perinatal outcomes of indicated inductions of labor. *J Matern Fetal Neonatal Med* 2015; 1–5. 10.3109/14767058.2015.1085966 26372677

[24] Gao Y Xue Q Chen G An analysis of the indications for cesarean section in a teaching hospital in China. *Eur J Obstet Gynecol Reprod Biol* 2013; 170:414–418. 23978503 10.1016/j.ejogrb.2013.08.009

[25] Beucher G Viaris de Lesegno B Dreyfus M Maternal outcome of gestational diabetes mellitus. *Diabetes Metab* 2010; 36 (6 Pt 2):522–537. 21163418 10.1016/j.diabet.2010.11.006

[26] Chung SD Chen YH Keller JJ Urinary calculi increase the risk for adverse pregnancy outcomes: a nationwide study. *Acta Obstet Gynecol Scand* 2013; 92:69–74. 23017095 10.1111/aogs.12016

[27] Hung HW Yang PY Yan YH Increased postpartum maternal complications after cesarean section compared with vaginal delivery in 225 304 Taiwanese women. *J Matern Fetal Neonatal Med* 2016; 29:1665–1672. 26135778 10.3109/14767058.2015.1059806

[28] MacEvilly M Buggy D Back pain and pregnancy: a review. *Pain* 1996; 64:405–414. 8783303 10.1016/0304-3959(95)00184-0

[29] MacArthur C Lewis M Knox EG Investigation of long term problems after obstetric epidural anaesthesia. *BMJ* 1992; 304:1279–1282. 1535010 10.1136/bmj.304.6837.1279 PMC1881855

[30] Alexander JT McCormick PC Pregnancy and discogenic disease of the spine. *Neurosurg Clin N Am* 1993; 4:153–159. 8428151

[31] Frymoyer JW Back pain and sciatica. *N Engl J Med* 1988; 318:291–300. 2961994 10.1056/NEJM198802043180506

[32] Wolter T Szabo E Becker R Chronic low back pain: course of disease from the patient's perspective. *Int Orthop* 2011; 35:717–724. 20623120 10.1007/s00264-010-1081-x PMC3080499

[33] Russell R Groves P Taub N Assessing long term backache after childbirth. *BMJ* 1993; 306:1299–1303. 8518569 10.1136/bmj.306.6888.1299 PMC1677713

[34] Green LW Manual for scoring socioeconomic status for research on health behavior. *Public Health Rep* 1970; 85:815–827. 4989476 PMC2031767

[35] Scott DB Hibbard BM Serious non-fatal complications associated with extradural block in obstetric practice. *Br J Anaesth* 1990; 64:537–541. 2354090 10.1093/bja/64.5.537

[36] Sage DJ Epidurals, spinals and bleeding disorders in pregnancy: a review. *Anaesth Intensive Care* 1990; 18:319–326. 2221324 10.1177/0310057X9001800307

[37] Schmidt A Nolte H Subdural and epidural hematomas following epidural anesthesia. A literature review. *Der Anaesthesist* 1992; 41:276–284. 1616118

[38] Mense S Nociception from skeletal muscle in relation to clinical muscle pain. *Pain* 1993; 54:241–289. 8233542 10.1016/0304-3959(93)90027-M

[39] Gronblad M Liesi P Korkala O Innervation of human bone periosteum by peptidergic nerves. *Anat Rec* 1984; 209:297–299. 6205609 10.1002/ar.1092090306

[40] Madeira HG Garcia JB Lima MV Disability and factors associated with gestational low back pain. *Rev Bras Ginecol Obstet* 2013; 35:541–548. 24500508 10.1590/s0100-72032013001200003

[41] Fast A Shapiro D Ducommun EJ Low-back pain in pregnancy. *Spine* 1987; 12:368–371. 2956698 10.1097/00007632-198705000-00011

[42] Ostgaard HC Andersson GB Previous back pain and risk of developing back pain in a future pregnancy. *Spine* 1991; 16:432–436. 1828628 10.1097/00007632-199104000-00008

[43] Gutke A Ostgaard HC Oberg B Predicting persistent pregnancy-related low back pain. *Spine* 2008; 33:E386–E393. 18496334 10.1097/BRS.0b013e31817331a4

[44] To WW Wong MW Factors associated with back pain symptoms in pregnancy and the persistence of pain 2 years after pregnancy. *Acta Obstet Gynecol Scand* 2003; 82:1086–1091. 14616251 10.1046/j.1600-0412.2003.00235.x

[45] Chen YL Chang Y Yeh YL Timing of epidural analgesia intervention for labor pain in nulliparous women in Taiwan: a retrospective study. *Acta Anaesthesiol Taiwan* 2013; 51:112–115. 24148739 10.1016/j.aat.2013.09.001

[46] Hung TH Hsieh TT Liu HP Differential effects of epidural analgesia on modes of delivery and perinatal outcomes between nulliparous and multiparous women: a retrospective cohort study. *PloS One* 2015; 10:e0120907. 25807240 10.1371/journal.pone.0120907 PMC4373716

[47] Chang KY Chan KH Chang SH Decision analysis for epidural labor analgesia with Multiattribute Utility (MAU) model. *Clin J Pain* 2008; 24:265–272. 18287834 10.1097/AJP.0b013e31816111a5

[48] Huang CH Hsieh YJ Wei KH A comparison of spinal and epidural anesthesia for cesarean section following epidural labor analgesia: a retrospective cohort study. *Acta Anaesthesiol Taiwan* 2015; 53:7–11. 25736588 10.1016/j.aat.2015.01.003

[49] Zhang J Yancey MK Klebanoff MA Does epidural analgesia prolong labor and increase risk of cesarean delivery? A natural experiment. *Am J Obstet Gynecol* 2001; 185:128–134. 11483916 10.1067/mob.2001.113874

[50] Carvalho B Wang P Cohen SE A survey of labor patient-controlled epidural anesthesia practice in California hospitals. *Int J Obstet Anesth* 2006; 15:217–222. 16798447 10.1016/j.ijoa.2006.03.006

[51] Khor LJ Jeskins G Cooper GM National obstetric anaesthetic practice in the UK 1997/1998. *Anaesthesia* 2000; 55:1168–1172. 11121925 10.1046/j.1365-2044.2000.01720.x

